# A Transgenic Rat for Specifically Inhibiting Adult Neurogenesis^1^,^2^,^3^

**DOI:** 10.1523/ENEURO.0064-16.2016

**Published:** 2016-05-31

**Authors:** Jason S. Snyder, Laura Grigereit, Alexandra Russo, Désirée R. Seib, Michelle Brewer, James Pickel, Heather A. Cameron

**Affiliations:** 1 Section on Neuroplasticity, National Institute of Mental Health, National Institutes of Health, Bethesda, Maryland 20892; 2 Transgenic Core Facility, National Institute of Mental Health, National Institutes of Health, Bethesda, Maryland 20892, and; 3 Department of Psychology, Djavad Mowafaghian Centre for Brain Health, University of British Columbia, Vancouver, BC, V6T 1Z4, Canada

**Keywords:** adult neurogenesis, hippocampus, olfactory bulb, plasticity

## Abstract

The growth of research on adult neurogenesis and the development of new models and tools have greatly advanced our understanding of the function of newborn neurons in recent years. However, there are still significant limitations in the ability to identify the functions of adult neurogenesis in available models. Here we report a transgenic rat (TK rat) that expresses herpes simplex virus thymidine kinase in GFAP+ cells. Upon treating TK rats with the antiviral drug valganciclovir, granule cell neurogenesis can be completely inhibited in adulthood, in both the hippocampus and olfactory bulb. Interestingly, neurogenesis in the glomerular and external plexiform layers of the olfactory bulb was only partially inhibited, suggesting that some adult-born neurons in these regions derive from a distinct precursor population that does not express GFAP. Within the hippocampus, blockade of neurogenesis was rapid and nearly complete within 1 week of starting treatment. Preliminary behavioral analyses indicate that general anxiety levels and patterns of exploration are generally unaffected in neurogenesis-deficient rats. However, neurogenesis-deficient TK rats showed reduced sucrose preference, suggesting deficits in reward-related behaviors. We expect that TK rats will facilitate structural, physiological, and behavioral studies that complement those possible in existing models, broadly enhancing understanding of the function of adult neurogenesis.

## Significance Statement

Adult neurogenesis contributes to the physiological and behavioral functions of the brain, but our understanding is subject to the limitations of available animal models. Although mice have been invaluable research models, their small size and limited behavioral repertoire hinders certain types of experiments. We have therefore created a transgenic rat in which neurogenesis can be inhibited completely and selectively in adulthood in two brain regions, the hippocampus and subventricular zone-olfactory bulb. We expect that these rats will complement existing approaches and enhance understanding of the function of adult neurogenesis.

## Introduction

Although the existence of adult hippocampal neurogenesis is no longer disputed, its function is still uncertain. Additional anatomical, behavioral, and physiological studies are needed to understand how new neurons affect circuits and cognition. Historically, most rodent neuroscience studies were done in rats. However, increased focus on genetics and the development of transgenic technology have tipped the balance strongly in favor of mice. Nonetheless, rat studies may be increasing again due to an increase in sequence data as well as recognition that greater similarity to humans in some genes, such as those encoding tau proteins, can lead to transgenic rat models that better replicate the spectrum of human pathology (Abbott, 2004; Cohen et al., 2013).

Their large body size, relative to mice, has long made rats the model of choice for physiology studies (Abbott, 2004). This is equally true for neurophysiology: nearly all of the Nobel-prize winning place cell work, for example, was done in rats (Moser et al., 2008). This is due in large part to size, which gives rats a better ability to support large multielectrode arrays and investigators greater accuracy in stereotaxic targeting. However, hippocampal place cell representations are also less stable in mice than in rats, which can make it challenging to relate firing patterns to environmental inputs and ongoing behavior (Kentros et al., 2004). Understanding the function of new hippocampal neurons at the circuit level will almost certainly be advanced by studying the network consequences of altering neurogenesis in rats.

Advantages in behavioral testing may provide the greatest motivation for developing models of altered adult neurogenesis in rats. Thus far, the majority of behavioral studies of hippocampal neurogenesis have focused on spatial or episodic-like memory and innate emotional behaviors. However, early studies, as well as more recent findings implicate the hippocampus, and therefore potentially new dentate gyrus neurons, in a number of additional behaviors including orienting and attention, behavioral inhibition, delay-discounting, and salience attribution (Lodge and Grace, 2011; Cameron and Glover, 2015). Most of these behaviors have been studied extensively in rats and very little in mice, so testing the role of neurogenesis in these behaviors will almost certainly be more straightforward in rats. Cognitive scientists in general are increasingly embracing the rat as an ideal behavioral test subject for a broad spectrum of behavioral studies, arguing that they are capable of more complex tasks than mice but with greater potential for genetic manipulation than nonhuman primates (Abbott, 2004).

Options for manipulating neurogenesis in rats currently exist: chemical, radiological, and viral strategies have all been successfully used to inhibit adult neurogenesis (Tada et al., 2000; Shors et al., 2001; Jessberger et al., 2009). However, the advantages of transgenic lines, which include the ability to eliminate neurogenesis noninvasively, are largely limited to mice. To address this problem, we have developed a transgenic rat in which neurogenesis can be specifically inhibited, either partially or completely, at any age. The approach uses a well characterized pharmacogenetic strategy through which cell-specific expression of the simplex virus thymidine kinase (HSV-TK) gene enables selective ablation of proliferating cells. HSV-TK (but not endogenous mammalian TK) phosphorylates nucleoside analogs such as ganciclovir, which are then incorporated into the DNA of replicating cells, leading to inhibition of DNA synthesis and apoptosis (Elion et al., 1977; Furman et al., 1980). Depending on the TK expression pattern and the timing of ganciclovir administration, specific populations of precursor cells that are active at various stages of development can be selectively ablated (Borrelli et al., 1989; Heyman et al., 1989; Delaney et al., 1996). This approach has been used to inhibit neurogenesis in adult mice (Garcia et al., 2004; Saxe et al., 2006; Deng et al., 2009; Singer et al., 2009; Snyder et al., 2011; Cummings et al., 2014; Seo et al., 2015). It has recently been tried in rats as well, though with limited success, likely related to random insertion effects (Groves et al., 2013). Here, we describe a new transgenic rat strain that shows HSV-TK expression under the control of the glial fibrillary acidic protein (GFAP) promoter and ganciclovir-induced loss of adult neurogenesis in two regions that generate new neurons from GFAP-expressing radial cells: the dentate gyrus and subventricular zone-olfactory bulb. Initial behavioral analyses indicate that neurogenesis-deficient GFAP-TK rats are similar to neurogenesis-intact wild-type littermates in general locomotion and baseline anxiety but display reduced sucrose preference, suggesting alterations in reward-related behaviors. We expect that these rats, and rat models in general, will complement existing mouse models and facilitate understanding of the functions of adult neurogenesis.

## Methods

All procedures followed the Institute of Laboratory Animal Research guidelines and were approved by the Animal Care and Use Committee of the National Institute of Mental Health.

### Transgenic animals

Transgenic rats expressing HSV-TK under the human GFAP promoter (GFAP-TK, or TK rats) were generated on a Long–Evans background using the pGfa2-TK1 plasmid (Delaney et al., 1996) applying standard techniques. Female rats were used for breeding, as males of this strain fail to pass on the transgene, as is commonly seen in lines that express HSV-TK under any promoter (Braun et al., 1990; al-Shawi et al., 1991; Delaney et al., 1996). Male rats were used for all experiments. Rats were weaned at 21–28 d of age, pair-housed, and genotyped by PCR. Rats were subjected to a 12 h light/dark schedule with lights on at 6:00 A.M. Neurogenesis was suppressed by using the orally available prodrug, valganciclovir, which is enzymatically converted to ganciclovir. For initial determination of valganciclovir efficacy, rats were given 7.5 mg valganciclovir on weekdays (ie, 5 d on, 2 d off) for 8 weeks beginning at 8 weeks of age. Valganciclovir was delivered in a 0.5 g pellet of a 1:1 mixture of ground chow and peanut butter. To minimize neophobia, rats were exposed to the chow–peanut butter mixture in their home cage for several days prior to treatment. On treatment days, individual valganciclovir pellets were specifically given to each rat in the cage by hand to ensure consistent dosing. For behavioral experiments, rats were treated on weekdays for 3 weeks (at which point neurogenesis was found to be maximally reduced) and then treated only twice per week (Monday and Thursday) for the remainder of the experiment. This reduced treatment protocol effectively prevented recovery of neurogenesis while minimizing unnecessary costs and time spent treating rats. We refer to valganciclovir-treated wild-type (WT) and TK rats as v-WT and v-TK rats. These rats will be made available to other investigators through the Rat Resource and Research Center.

### Immunohistochemical analyses

To determine the temporal onset of neurogenesis inhibition, WT and TK rats were treated with valganciclovir for 8 weeks, and each rat was injected once with bromodeoxyuridine (BrdU, 200 mg/kg, i.p.) on the 5th, 10th, 15th, or 20th day of valganciclovir treatment (ie, weeks 1, 2, 3, or 4). At the end of the 8 weeks, all rats were perfused with 4% paraformaldehyde and brains were collected. Coronal sections (40 µm) of the olfactory bulb (unilateral) and hippocampus (bilateral) were cut on a freezing sliding microtome and processed for immunohistochemical analyses. Briefly, to quantify adult-born neurons, a 1 in 12 series of sections through the entire dentate gyrus and entire olfactory bulb was immunostained using a mouse anti-BrdU primary antibody (BD Biosciences), biotinylated goat anti-mouse secondary antibody (Sigma-Aldrich), and peroxidase-DAB detection. Cells were counted throughout the entire series to provide stereological estimates. For assessing the phenotype of BrdU+ cells, sections were double-labeled with rat anti-BrdU (Accurate) and mouse anti-NeuN (Millipore) primary antibodies and AlexaFluor secondary antibodies. Approximately 50 BrdU+ cells were examined, and the proportion that exhibited NeuN immunoreactivity, a marker of neuronal phenotype, was multiplied by the total number of (peroxidase-DAB stained) BrdU+ cells to estimate the total number of labelled adult-born neurons in v-WT and v-TK rats. GFAP+ astrocytes and radial glial cells were identified with goat anti-GFAP antibody (Santa Cruz Biotechnology) and rabbit anti-HSV-TK antibody (a gift from GlaxoSmithKline). In a separate experiment, rats were treated with valganciclovir for 8 weeks and perfused 0, 4, or 8 weeks after stopping treatment to determine whether neurogenesis recovers. Here, immature neurons were identified by immunostaining with goat anti-doublecortin (DCX) antibody (Santa Cruz Biotechnology) and quantified from a 1 in 12 series of sections through the entire dentate gyrus.

### Behavioral testing

Rats were treated with valganciclovir for 6–8 weeks before undergoing behavioral testing. Different cohorts of rats were used for each test. Open field: rats were placed in an open field (white plastic box, 50 × 50 × 50 cm) for 15 mins and the distance travelled and time spent in concentric outer, middle and center 10-cm-wide zones, measures of general locomotion and anxiety-related behavior, were calculated by Ethovision software (Noldus). To encourage exploration of the center/anxiogenic portion of the open field, a small toy was secured to the floor of the center of the open field (metal wire cylinder filled with marbles, ∼ 3 cm wide × 5 cm high). Novelty-suppressed feeding: rats were food deprived for 24 h prior to testing. On the test day, rats were placed in the novel open field (the same one used for open-field testing) with five pellets of rodent chow present in the center. The latency to begin consuming the food, which is reduced by antidepressants and anxiolytic drugs (Bodnoff et al., 1988, 1989) and modulated by adult neurogenesis in mice (Santarelli et al., 2003; Snyder et al., 2011), was recorded as a measure of innate anxiety. Sucrose preference: rats were given two water bottles containing water and a 1% sucrose solution (location of each bottle counterbalanced across cages). Preference for the sucrose solution was measured by weighing the bottles before and after 4 d of constant access to the water bottles. Immediately after this measure, the water bottles were removed for 8 h, and placed back in the same location for a 10 min preference test during the early portion of the dark/active cycle (∼7:00–8:00 P.M.).

## Results

**Figure 1.**
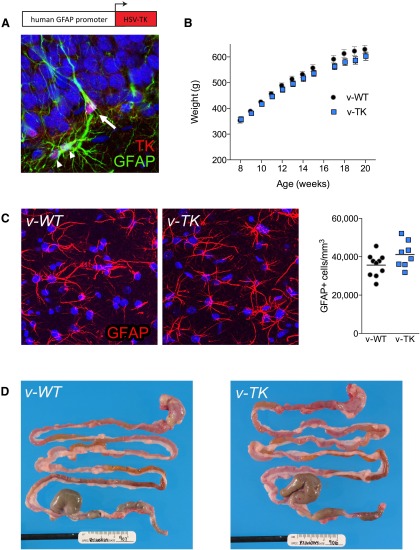
The GFAP-TK rat model. ***A***, Directing HSV-TK expression to GFAP+ cells led to TK+ radial precursor cells (arrow) and TK+ stellate astrocytes (arrowheads) in transgenic rats. ***B***, Following valganciclovir treatment beginning at 8 weeks of age (7.5 mg/d, weekdays), v-TK rats gained weight more slowly than v-WT rats (*n* = 11–12; genotype×age repeated-measures ANOVA: genotype, *p* = 0.26 ; age, *p* < 0.0001; interaction, *p* = 0.002), though multiple comparisons revealed that v-WT and v-TK rats did not differ significantly at any age. Weights were not included at 16 weeks of age, because rats were food restricted for testing of novelty suppressed feeding. Symbols indicate mean ± SEM. ***C***, The density of astrocytes in the molecular layer of the dentate gyrus was not different between v-WT and v-TK rats after 10 weeks of treatment with valganciclovir (*p* = 0.08). Symbols represent data from individual rats; lines indicate group mean values. ***D***, The gastrointestinal tracts of v-WT and v-TK rats were healthy and indistinguishable from each other following 9 weeks of treatment with valganciclovir.

Driving HSV-TK expression with the human GFAP promoter led to clear TK immunostaining in GFAP+ astrocytes, as well as GFAP+ radial glial stem cells of the hippocampus (Seri et al., 2001; Fig. 1*A*). Following treatment with valganciclovir (7.5 mg/d, 5 d/week), v-TK rats appeared generally healthy and gained weight, but weighed slightly (7%) less than v-WT rats after prolonged treatment (genotype × time interaction, *p* < 0.01; Fig. 1*B*). Treatment with valganciclovir for 8 weeks did not affect numbers of GFAP+ astrocytes in the dentate gyrus molecular layer (Fig. 1*C*) or the appearance of the GI tract (Fig. 1*D*), consistent with previous findings in v-TK mice (Snyder et al., 2011), suggesting that postmitotic GFAP+ cells are not impacted by the TK transgene.

### Effective inhibition of adult neurogenesis in the dentate gyrus

**Figure 2.**
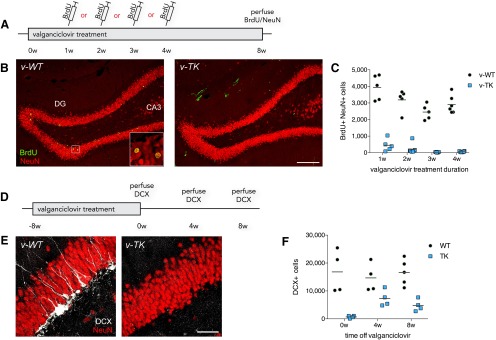
Inhibition of dentate gyrus neurogenesis in GFAP-TK rats. ***A***, Timeline for inhibition experiment. ***B***, Confocal images illustrating adult-born BrdU+NeuN+ cells in v-WT rats and their absence in v-TK rats that were injected with BrdU after 4 weeks of valganciclovir treatment. Scale bar, 200 µm. ***C***, Quantitative analyses of neurogenesis blockade revealed that neurogenesis was greatly reduced after 1 week of valganciclovir treatment and nearly completely eliminated with additional treatment (genotype × treatment duration ANOVA: genotype and treatment duration effects both *p* < 0.0001; interaction: *p* = 0.17). ***D***, Timeline for inhibition-recovery experiment. Doublecortin-positive (DCX+) cells were assessed after 8 weeks of valganciclovir treatment followed by 0, 4, or 8 weeks without valganciclovir treatment. ***E***, Confocal images illustrating DCX+ immature neurons in WT but not TK rats that were treated with valganciclovir for 8 weeks. Scale bar, 50 µm. ***F***, DCX+ cells were greatly reduced in TK rats (genotype effect: *p* < 0.0001). There was partial recovery after 4 weeks but no further change (genotype×time interaction: *p* = 0.0002; 4 and 8 week TK vs 0 week TK, *p* < 0.0001; 4 week TK vs 8 week TK, *p* = 0.2652. Stereological estimates are shown in graph but statistics were run on values after log-transformation to achieve homogeneity of variance. Symbols represent data from individual rats; lines indicate group mean values.

Eight-week-old WT and TK rats were treated with valganciclovir for 8 weeks and received BrdU injections to determine the extent of neurogenesis inhibition. BrdU was injected after either 1, 2, 3, or 4 weeks to determine the temporal onset of neurogenesis inhibition in TK rats, and rats were treated with valganciclovir for another 4 weeks, at which point all BrdU+ cells express strong levels of NeuN and have passed immature cell death stages (Dayer et al., 2003; Snyder et al., 2009). In v-WT rats, many BrdU+ cells were observed, and there was a modest reduction with age (Fig. 2*C*). In v-TK rats, however, there were significantly fewer BrdU+NeuN+ cells after 1 week of treatment (88% reduction) and even fewer of these cells at later time points (93% reduction at 2 weeks, 99% at 3 weeks, and 98% at 4 weeks; Fig. 2*C*). Because BrdU acts as a pulse label and could fail to detect transient recovery of neurogenesis in TK rats (for example, on weekends when valganciclovir was not administered), we also performed immunohistochemistry for DCX, which is expressed in a large proportion of neurons born during the 4 weeks prior to death (Brown et al., 2003; Snyder et al., 2009). Qualitative analysis revealed near complete loss of DCX+ cells in v-TK brains, indicating that ablation of neurogenesis was complete (Fig. 2*F*). This marker, therefore, provides a simple method for validating the genotype and valganciclovir effectiveness in each rat. After treating 8-week-old WT and TK rats with valganciclovir for 8 weeks, drug was discontinued for 4 or 8 weeks to investigate the recovery of adult neurogenesis. DCX+ cell number partially recovered after 4 weeks without valganciclovir but remained at 30% of the WT value after 8 weeks post-valganciclovir (Fig. 2*F*).

### Effective inhibition of adult neurogenesis in the olfactory bulb

**Figure 3.**
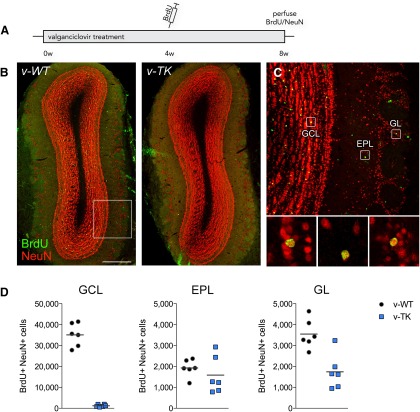
Inhibition of adult neurogenesis in the olfactory bulb. ***A***, Experimental timeline. ***B***, Confocal images illustrating adult-born BrdU+NeuN+ cells in v-WT rats but not v-TK rats. ***C***, Insets (left to right) show BrdU+NeuN+ neurons in the granule cell layer (GCL), external plexiform layer (EPL), and glomerular layer (GL). ***D***, Four weeks of valganciclovir treatment reduced neurogenesis by 95% in the granule cell layer (*p* < 0.0001), did not significantly impact neurogenesis in the external plexiform layer (15% reduction, *p* = 0.4), and reduced neurogenesis by 48% in the glomerular layer (*p* = 0.002). Symbols represent data from individual rats; lines indicate group mean values.

The subventricular zone (SVZ) also contains neurogenic, GFAP+ radial glia precursors (Doetsch et al., 1999; Morshead et al., 2003; Garcia et al., 2004). These cells give rise in adulthood to neurons that migrate to the olfactory bulb, where they modify circuits and olfactory behaviors (Lepousez et al., 2013; Cummings et al., 2014). To determine whether olfactory neurogenesis was disrupted in GFAP-TK rats, we quantified BrdU+NeuN+ cells in the olfactory bulb of rats that received BrdU after 4 weeks of valganciclovir treatment and survived for an additional 4 weeks, a time point at which all BrdU-labeled cells will have exited the rostral migratory stream (Lemasson et al., 2005). v-TK rats had a 95% reduction in BrdU+NeuN+ cells in the granule cell layer of the olfactory bulb, the primary target of SVZ neurogenesis (Fig. 3). A smaller number of physiologically distinct neurons are added to other regions of the olfactory bulb. Neurogenesis in these regions was less affected in GFAP-TK rats: BrdU+NeuN+ periglomerular neurons were reduced by only 51% in v-TK rats (*p* = 0.002), and no significant decrease was observed in the external plexiform layer (*p* = 0.4; Fig. 3).

### Normal open-field behavior in v-TK rats

**Figure 4.**
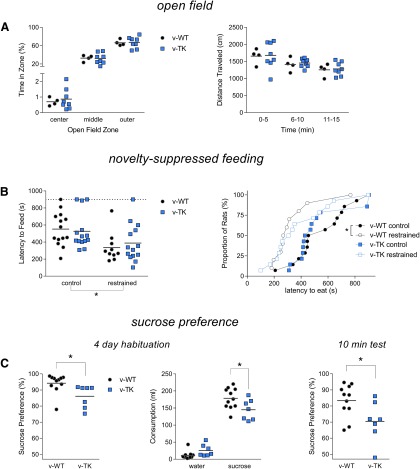
GFAP-TK rat behavior. ***A***, v-WT and v-TK rats displayed similar behavior in the open field. Both genotypes spent progressively more time in the outer portions of the open field, near the walls [left graph; genotype×zone repeated-measures (RM) ANOVA; effect of genotype: *p* = 0.7; effect of zone: *p* < 0.0001; interaction: *p* = 1]. Exploratory behavior also was not different between genotypes, with both v-WT and v-TK rats travelling less in successive 5 min bins during the 15 min test (right graph; genotype×time RM ANOVA; effect of genotype: *p* = 0.8; effect of time: *p* < 0.001; interaction: *p* = 1). ***B***, Anxiety-related behavior in the novelty-suppressed feeding test was not significantly difference between v-WT and v-TK rats. Both groups displayed reduced latency to consume food following 30 min restraint stress compared with unrestrained controls (left graph; genotype×restraint ANOVA; effect of genotype: *p* = 0.6, effect of restraint: *p* < 0.0001, interaction: *p* = 0.7). Right graph illustrates the distribution of consumption latencies. Restraint significantly shifted the distribution in v-WT rats but not v-TK rats (log rank test of all 4 curves: *p*=0.04; v-WT comparison: *p*=0.02; v-TK comparison: *p*=0.2). ***C***, Left graph, v-TK rats had a reduced preference for sucrose solution over water as measured by sucrose consumption as a percentage of total consumption over 4 d (*p* = 0.02). Middle graph, A genotype×solution RM ANOVA revealed that TK rats consumed less sucrose solution but not significantly more water (effect of genotype: *p* = 0.3; effect of solution: *p* < 0.0001; interaction: *p* = 0.007; *post hoc* v-WT vs v-TK water: *p* = 0.4 and sucrose: *p* = 0.01). Right graph, v-TK rats also displayed reduced sucrose preference during a 10 min preference test (*p*=0.03).

Following 6 weeks of valganciclovir treatment, general activity and anxiety levels were assessed in v-WT and v-TK rats in an open-field test with a novel object in the center. Innate anxiety-related behavior was examined by quantifying time spent in concentric inner, middle and outer zones of the open field. v-WT and v-TK rats explored the open field similarly, spending the majority of the trial in the outer regions of the open field, near the wall (Fig. 4*A*). v-WT and v-TK rats also traveled equivalent distances, covering progressively less territory as they habituated to the environment over the course of the 15 min test (Fig. 4*A*).

### Normal behavior in the novelty-suppressed feeding paradigm in v-TK rats

Adult neurogenesis in mice has been shown to regulate anxiety/depression-related behavior in the novelty-suppressed feeding test, where food-deprived mice are placed in an anxiogenic novel environment and are tested for their latency to begin consuming food. Neurogenesis does not affect baseline performance in this task, but is required for the effects of antidepressants and minimizes the effects of stress on behavior in this paradigm (Santarelli et al., 2003; Snyder et al., 2011). We therefore tested naive rats as well as rats that were exposed to a 30 min restraint session immediately prior to the test. v-WT and v-TK rats did not differ at baseline or following restraint when analyzed by ANOVA, in contrast to previous studies in mice (Snyder et al., 2011). However, restraint significantly reduced the latency to begin consuming food in both v-WT and v-TK rats, also in contrast to its effects in mice (Fig. 4*B*). We also performed nonparametric analyses of the survival/distribution curves because not all of the groups passed tests of normality (specifically the restrained v-WT group). While restraint significantly shifted the distribution of feeding latencies in v-WT rats it had no significant effect on v-TK rats (Fig. 4*B*; Table 1).

**Table 1.** Statistical table

	**Figure**	**Description**	**Data structure**	**Type of test**	**Exact *p* value**
a	1*B*	Weights-effect of genotype	Normal distribution	Two-way repeated-measures ANOVA	*p* = 0.26
b	1*B*	Weights-effect of age	Normal distribution	Two-way repeated-measures ANOVA	*p* < 0.0001
c	1*B*	Weights-genotype × age interaction	Normal distribution	Two-way repeated-measures ANOVA	*p* = 0.002
d	1*C*	GFAP+ cells	Normal distribution	*t* test	*p* = 0.08
e	2*C*	BrdU+ cells in DG: effect of genotype	Normal distribution	Two-way ANOVA	*p* < 0.0001
f	2*C*	BrdU+ cells in DG: effect of treatment duration	Normal distribution	Two-way ANOVA	*p* < 0.0001
g	2*C*	BrdU+ cells in DG: genotype × treatment duration interaction	Normal distribution	Two-way ANOVA	*p* = 0.17
h	2*F*	DCX+ cells in DG: effect of genotype	Assumed normal distribution	Two-way ANOVA	*p* < 0.0001
i	2*F*	DCX+ cells in DG: genotype × recovery period interaction	Assumed normal distribution	Two-way ANOVA	*p* = 0.0002
j	2*F*	DCX+ cells in DG-TK 4 week and 8 week recovery vs 0 week	Assumed normal distribution	Sidak *post hoc* tests	*p* < 0.0001
k	2*F*	DCX+ cells in DG-TK 4 week recovery vs TK 8 week recovery	Assumed normal distribution	Sidak *post hoc* test	*p* = 0.2652
l	3*D*	BrdU+ cells in olfactory bulb-GCL	Normal distribution	*t* test	*p* < 0.0001
m	3*D*	BrdU+ cells in olfactory bulb-EPL	Normal distribution	*t* test	*p* = 0.4
n	3*D*	BrdU+ cells in olfactory bulb-GL	Normal distribution	*t* test	*p* = 0.002
o	4*A*	Open-field behavior test-zones: effect of genotype	Assumed normal distribution	Two-way repeated-measures ANOVA	*p* = 0.7
p	4*A*	Open-field behavior test-zones: effect of zone	Assumed normal distribution	2 way repeated measures ANOVA	*p* < 0.0001
q	4*A*	Open-field behavior test-zones: genotype × zone interaction	Assumed normal distribution	Two-way repeated-measures ANOVA	*p* = 1
r	4*A*	Open-field behavior test-distance traveled: effect of genotype	Assumed normal distribution	Two-way repeated-measures ANOVA	*p* = 0.8
s	4*A*	Open-field behavior test-distance travelled: effect of time	Assumed normal distribution	Two-way repeated-measures ANOVA	*p* < 0.001
t	4*A*	Open-field behavior test-distance traveled: genotype × time interaction	Assumed normal distribution	Two-way repeated-measures ANOVA	*p* = 1
u	4*B*	Novelty suppressed feeding test-latency: effect of genotype	Normal and potentially non-normal groups	Two-way ANOVA	*p* = 0.6
v	4*B*	Novelty suppressed feeding test-latency: effect of restraint	Normal and potentially non-normal groups	Two-way ANOVA	*p* < 0.0001
w	4*B*	Novelty suppressed feeding test-latency: genotype × restraint interaction	Normal and potentially non-normal groups	Two-way ANOVA	*p* = 0.7
x	4*B*	Novelty suppressed feeding test-latency: distribution of feeding latencies (survival analyses)	Normal and potentially non-normal groups	Log rank test (Mantel–Cox)	*p* = 0.04
y	4*B*	Novelty suppressed feeding test-latency: distribution of feeding latencies (survival analyses): v-WT control vs v-WT restraint	Normal and potentially non-normal groups	Log rank test (Mantel–Cox)	*p* = 0.02
z	4*B*	Novelty suppressed feeding test-latency: distribution of feeding latencies (survival analyses): v-TK control vs v-TK restraint	Normal and potentially non-normal groups	Log rank test (Mantel–Cox)	*p* = 0.2
aa	4*C*	Sucrose preference-habituation preference	Normal distribution	*t* test	*p* = 0.02
bb	4*C*	Sucrose preference-habituation consumption: effect of genotype	Normal distribution	Two-way repeated-measures ANOVA	*p* = 0.3
cc	4*C*	Sucrose preference-habituation consumption: effect of solution	Normal distribution	Two-way repeated-measures ANOVA	*p* < 0.0001
dd	4*C*	Sucrose preference-habituation consumption: interaction	Normal distribution	Two-way repeated-measures ANOVA	*p* = 0.007
ee	4*C*	Sucrose preference-habituation consumption-v-WT vs v-TK water	Normal distribution	Two-way repeated-measures ANOVA with Sidak *post hoc*	*p* = 0.4
ff	4*C*	Sucrose preference-habituation consumption: v-WT vs v-TK sucrose	Normal distribution	Two-way repeated-measures ANOVA with Sidak *post hoc*	*p* = 0.01
gg	4*C*	Sucrose preference acute 10 min test	Normal distribution	*t* test	*p* = 0.03

### v-TK rats show reduced sucrose preference

To examine whether loss of adult neurogenesis leads to anhedonia-like behavior, v-WT and v-TK rats were given free access to both water and a 1% sucrose solution. Consistent with our previous findings in v-TK mice (Snyder et al., 2011), we found that v-TK rats showed reduced preference for sucrose solution. However, whereas the sucrose preference deficit in mice required switching the locations of the two solutions, v-TK rats showed reduced sucrose preference when the sucrose and water bottle locations remained constant during a 4 d habituation period and an acute 10 min preference test that followed 8 h of water deprivation (Fig. 4*C*).

## Discussion

We have generated a GFAP-TK rat line and found that neurogenesis in the dentate gyrus can be completely inhibited in these rats by oral treatment with valganciclovir. Blockade of neurogenesis was nearly complete within 1 week of starting treatment. If treatment was stopped after 8 weeks, neurogenesis partially recovered over the next 4 weeks but remained low after 8 weeks without valganciclovir. Generation of olfactory bulb granule cells was also completely inhibited by valganciclovir in these GFAP-TK rats. In contrast, neurogenesis in the glomerular and external plexiform layers of the olfactory bulb was only partially inhibited, suggesting that some adult-born neurons in these layers derive from a different precursor population that does not express GFAP. Initial behavioral analyses indicate that general anxiety levels and patterns of exploration are largely unaffected in neurogenesis-deficient rats. However, v-TK rats showed reduced sucrose preference and a small decrease in weight gain, suggesting effects on reward-related behaviors.

### Specificity of the model

A concern with any ablation model is the possibility of nonspecific cell killing. The HSV-TK approach was designed such that viral TK expression, exogenous delivery of the nucleoside analog ganciclovir, or prodrug valganciclovir, and cell division are all required for cell toxicity. HSV-TK, unlike mammalian TK, phosphorylates ganciclovir, which becomes incorporated into DNA and stops further replication, killing the cell (Borrelli et al., 1988; Heyman et al., 1989). The drug, therefore, provides temporal specificity, whereas both the GFAP promoter and the requirement for cell division provide cell-type specificity. Although astrocytes express GFAP, they are generated during the early postnatal period (Sauvageot and Stiles, 2002; Petrik et al., 2013) and do not normally divide in adulthood (Amat et al., 1996; Burns et al., 2009). They are not, therefore, expected to be vulnerable to valganciclovir in adulthood in the GFAP-TK model, consistent with evidence from healthy GFAP-TK mice (Bush et al., 1998; Snyder et al., 2011). To test whether mature astrocytes were killed in our model, we quantified GFAP+ stellate astrocytes in the dentate gyrus of v-TK rats. We observed no loss of astrocytes in v-TK rats and comparable morphology between v-WT and v-TK rats, indicating that ablation is specific to dividing GFAP+ cells, ie, radial glial cells. It is worth noting, however, that astrocytogenesis may occur following injury and be inhibited in GFAP-TK mice (Amat et al., 1996; Bush et al., 1999; Faiz et al., 2015; but see Burns et al., 2009). Thus, the GFAP-TK rat may be a useful tool for identifying functions of adult-generated astrocytes in brain repair, though this has not yet been investigated. Finally, a previous GFAP-TK mouse model has identified an essential role for GFAP+ enteric glial cells in gastrointestinal health (Bush et al., 1998), though not all GFAP-TK mouse lines display gut-related illness upon treatment with valganciclovir (Snyder et al., 2011). We found no intestinal inflammation, hemorrhage, or other gastrointestinal pathology in v-TK rats. Although nonspecific side effects can never be completely ruled out, we find that GFAP-TK rats are healthy and generally comparable to WT littermates, making them an effective model for investigating functions of adult neurogenesis.

### Behavioral phenotype of GFAP-TK rats

We observed a small but significant difference in weight gain, with lower weights in v-TK rats after long valganciclovir treatment duration. The lack of GI pathology in these animals suggests that weight differences may be a downstream effect of reducing adult neurogenesis rather than an off-target effect. Weight loss was greater at higher doses (15 mg/kg/d, data not shown) possibly reflecting an effect on other brain regions or an effect on enteric glia that only occurs at high doses. However, a specific role for adult neurogenesis is consistent with evidence for decreased weight gain in a very different ablation model, cranial irradiation (Snyder et al., 2005). A role for the hippocampus in feeding behavior is supported by evidence that hippocampal lesions impair rats’ ability to inhibit appetitive responding to formerly food-predicting cues, increasing feeding frequency in food-related contexts and leading to weight gain (Tracy et al., 2001). Furthermore, increasing activity in the dentate gyrus–CA3 network suppresses feeding behavior through projections to the lateral septum and BNST (Sweeney and Yang, 2015). If immature adult-born neurons have a net inhibitory effect (Lacefield et al., 2012; Restivo et al., 2015; Drew et al., 2016), our data are consistent with these previous findings and suggest that blocking adult neurogenesis may inhibit feeding behavior by elevating hippocampal activity.

Like decreased weight gain, our observation that v-TK rats show reduced sucrose preference is consistent with a neurogenic role in motivation to consume calories. Reduced sucrose preference is generally viewed as a measure of anhedonia, or a motivational change specific to highly pleasurable activity. However, the reduced sucrose preference in combination with small but significant weight decrease observed in v-TK rats suggests that this behavioral change may reflect general appetitive changes rather than anhedonia per se. Interestingly, unlike v-TK rats, which showed reduced sucrose preference throughout the habituation and testing periods, v-TK mice were different from v-WT mice only during testing and only when the location of the sucrose and water was switched (Snyder et al., 2011). Interestingly, in that study v-TK mice also did not weigh less than v-WT mice, suggesting that roles for neurogenesis in reward and feeding-related behaviors may be weaker, or otherwise different, in mice relative to rats.

NSF behavior in v-TK rats was markedly different from in our previous study of v-TK mice (Snyder et al., 2011). In that study, restraint stress increased latency to feed in v-TK mice and had no effect on latency in v-WT mice (Snyder et al., 2011). Here, restraint had the opposite effect and reduced latency in both v-WT and v-TK rats. This decreased latency in both rat genotypes is similar to what has been observed in mice with a 30 min post-stress delay (Snyder et al., 2012), so the differential effects seen in v-TK mice and v-TK rats may reflect differences in the complex series of stress responses across species. Because the NSF feeding latencies were not normally distributed in all groups, we also tested whether restraint altered the distribution of latencies. These analyses revealed a restraint-induced shift in the survival curves of v-WT rats but not v-TK rats (Fig. 4*B*). Examining these curves, restraint uniformly shifted v-WT rats toward shorter feeding latencies. In contrast, in v-TK rats, restraint appears to reduce latencies in “early feeders” but not “late feeders”, suggesting that individual differences might dictate when neurogenesis regulates emotional behavior in rats. NSF behavior has been tested in an independently generated GFAP-TK rat, however, anxiety-like effects observed in untreated rats in this strain suggest a random gene insertion effect on anxiety that precludes interpretation of neurogenesis-dependent effects on neophagia (Groves et al., 2013).

The lack of a genotype difference in the open field in the current study is not surprising. A meta-analysis of elevated plus maze and open-field tests from 25 datasets found no evidence for an effect of adult neurogenesis in these innate anxiety tests. Meta analyses of contextual fear conditioning and spatial water maze behavior also found no significant spatial phenotype (Groves et al., 2013). Negative findings on spatial tasks, including contextual fear conditioning, spatial learning/reference memory in the water maze, spatial working memory in the radial maze, and behavioral pattern separation in the radial maze, in the Groves GFAP-TK rat also provide direct evidence, regardless of potential random insertion effects, that rats lacking adult neurogenesis can perform many spatial behaviors normally (Groves et al., 2013). However, several studies have reported spatial learning-related functions in neurogenesis-ablated animals under specific conditions (Drew et al., 2010; Burghardt et al., 2012). Given that adult neurogenesis affects emotion regulation (Revest et al., 2009; Snyder et al., 2011), it may be that differences in affective components of testing procedures contribute to this variability. Together, variability in cross-species and cross-laboratory comparisons of the effects of neurogenesis suggest that new neurons may not directly mediate anxiety, feeding, or spatial behavior but may instead alter other, as yet unidentified, cognitive processes involved in these behaviors.

### Strategies for manipulating neurogenesis

Elucidating the function of adult neurogenesis will require many complementary approaches; it is clear that there is no universally ideal model and that instead each approach presents it own advantages and limitations. A longstanding approach for causally testing behavioral functions of adult neurogenesis is the ablation model. This is essentially a cell-type-specific form of lesion, and was first performed with cytotoxic agents, such as MAM, AraC, and TMZ (Seri et al., 2001; Shors et al., 2001; Garthe et al., 2009) and irradiation (Parent et al., 1999; Madsen et al., 2003; Snyder et al., 2005). Chemical approaches, and potentially radiological approaches (provided that equipment is available), have the advantages of being relatively easy to administer and cost effective. However, even if they kill only dividing cells, they have the potential to affect non-neurogenic processes such as gliogenesis or peripheral cytogenesis, affecting immune, digestive, and other systems. Although central infusions or localized irradiation can minimize these off target effects, there are concerns about the specificity of these methods (Dupret et al., 2005). As an alternative, a number of transgenic mice have been developed in which neurogenesis can be conditionally suppressed in adulthood (Imayoshi et al., 2011). These include HSV-TK models that are designed to ablate Nestin+ and GFAP+ stem cells (Garcia et al., 2004; Saxe et al., 2006; Deng et al., 2009; Singer et al., 2009; Snyder et al., 2011) or DCX+ neuronal precursors (Seo et al., 2015), and mice that are engineered to express proapoptotic genes, such as Bax (Revest et al., 2009) or diptheria toxin (Imayoshi et al., 2008), in precursor cells and/or their progeny. Because any ablation model can potentially have off-target effects, an optimal strategy may be to employ multiple distinct approaches to confirm functions for new neurons (Saxe et al., 2006; Kitamura et al., 2009; Snyder et al., 2011).

Several new genetic methods have recently been developed to provide precise temporal control of effects on new neurons. Diphtheria toxin in combination with genetic expression of diphtheria toxin receptor, can be used to ablate adult-born neurons after they mature (Arruda-Carvalho et al., 2011). Retroviral or tamoxifen-inducible expression of light-sensitive ion channels or synthetic ligand-sensitive receptors in newborn neurons allows bidirectional control of new neuron activity on the order of milliseconds to hours with the ability to target specific cohorts of new neurons and reversibility that enables within-animal comparisons (Toni et al., 2008; Gu et al., 2012; Temprana et al., 2015). Ablation models that target neuronal precursors, including the GFAP-TK rat, lack this temporal precision. However, GFAP-TK transgenic lines have the advantage of allowing large numbers of animals to be treated rapidly and non-invasively, without the use of anesthetics or surgery, providing cell-type specificity through the GFAP promoter and temporal control via administration of valganciclovir. The virtually complete elimination of adult neurogenesis in this model allows for relatively straightforward interpretation of negative results without the possibility that stronger or more widespread inhibition would be effective. Inhibition of neurogenesis in the GFAP-TK rat, as in most ablation models, is largely maintained for several weeks after drug treatment is stopped. This can be a benefit, because the ganciclovir treatment can be stopped prior to behavior testing, though it hinders the ability to test for recovery of function. Another concern with chronic ablation is that interpretations may be confounded by compensatory effects. Decreased synaptic plasticity in a nestin-TK model is observed only transiently, presumably due to delayed compensatory plasticity from more mature neurons (Singer et al., 2011), though it is not clear whether this occurs in GFAP-TK mice or rats. Compensatory effects on behavior are also apparent in studies of hippocampal function, where lesions are thought to impact retrograde memory more than anterograde memory because other brain regions can be recruited for memory formation in the absence of a functional hippocampus (Maren et al., 1997; Moser and Moser, 1998; Wiltgen et al., 2006). Nevertheless, chronic ablation may be an appropriate model for long-term reductions in adult neurogenesis due to chronic stress, depression, and other neurological disorders.

We expect that the GFAP-TK rat will be valuable in several different types of studies where technical, historical, or biological concerns make rats more advantageous than mice. Head-mounted electrode arrays and miniature microscopes are better suited to the larger heads of rats than mice, and limited MRI resolution makes their larger brain better for imaging studies. GFAP-TK rats should therefore benefit studies of the role of adult neurogenesis in hippocampal and olfactory bulb circuitry and network properties. In addition, studies of the role of neurogenesis in several behavioral and disease models that have been developed in rats, eg, addiction and stroke models, should be able to move forward more quickly in GFAP-TK rats than in equivalent mice. Species differences in adult neurogenesis also suggest that additional investigation of neurogenesis function in rats will be of value. The maturation speed and survival rate of adult-born neurons is much greater in the rat than in the mouse (Snyder et al., 2009), suggesting that rats may show stronger neurogenesis-dependent behavior than mice. This enhanced neurogenesis in rats is seen in the neocortex, as well as the hippocampus (Snyder et al., 2009), suggesting that transgenic rats may also benefit the study of adult neurogenesis in so-called “non-neurogenic” regions such as the neocortex, striatum, hypothalamus, and circumventricular organs and allow it to move forward at a faster pace (Kokoeva et al., 2007; Cameron and Dayer, 2008; Migaud et al., 2010; Ohira et al., 2013; Furube et al., 2015; Inta et al., 2015). GFAP-TK rats should be useful for determining whether new neurons in these regions are generated from GFAP-expressing stem cells, like granule neurons, or from a separate precursor population as the current findings suggest is the case for some olfactory bulb interneuron populations. Finally, several studies point to species-specific responses of new neurons to experience; for example, adult-born hippocampal neurons in rats undergo dendritic plasticity (Tronel et al., 2010; Lemaire et al., 2012) and activity-dependent cell survival (Gould et al., 1999; Dupret et al., 2007; Epp et al., 2007) in response to spatial water maze training, both of which are less apparent in mice (Trinchero et al., 2015). Although there is clear evidence that adult neurogenesis is behaviorally significant in mice, species differences suggest that comparative studies will help to clarify the function of adult-born neurons by addressing their roles in interactions with the environment from multiple angles.

## Synthesis

The decision was a result of the Reviewing Editor Margaret McCarthy and the peer reviewers coming together and discussing their recommendations until a consensus was reached. A fact-based synthesis statement explaining their decision and outlining what is needed to prepare a revision is listed below. The following reviewer(s) agreed to reveal their identity: Cheryl Sisk

The major concern that must be addressed is whether the valganciclovir treatment is killing astrocytes throughout the brain as well as in the periphery, specifically the gut. The authors do not provide much discussion about potential off-target effects of the GFAP-TK plus vGCV procedure. The GFAP promoter will target not only adult neural progenitors, but also almost all astrocytes, which tile the entire CNS, as well as many other cells that express GFAP throughout the body, in peripheral nerves, in the gut and in multiple other organs. Transgenic GFAP-TK mice given high does of GCV develop a fatal gut illness (Bush et al Cell 93:189 (1998). This effect can be avoided by titrating the GCV dose. Have the present authors looked for or noticed such an effect? The authors do provide a figure on GFAP staining of hippocampal astrocytes which suggests no effect of the vGCV on them, but the authors do not explain or discuss why they are not concerned that there will be effects on astrocytes during their chronic treatment regimens, and that effects on astrocytes might impact on functional outcomes. Astrocytes are thought to be largely non-proliferative in healthy CNS, but the authors do not explain this or cite evidence for it. We ask that you thoroughly address this issue or provide additional data that speaks to it.

The second major concern focuses on the timing and dose of administration and how specific regimes were decided on. It will be useful to those seeking to adapt this model to understand thoroughly all of the twist and turns taken by the authors in establishing this important model. The authors seem to have adopted on/off delivery regimens of vGCV without explaining why. How did they arrive at their delivery regimen that starts out at 5 of 7 days per week and then shifts to 2 of 7 days per week? Why did they do this? Were there problems with continual dosing? Given that the vGCV is in the food, changing the food seems more work than simply leaving it. Changing the food also seems like it might lead to inconsistent dosing.

A third important question - would it be a good idea to include a statement in the manuscript somewhere (or maybe as a footnote) that these rats will be made available to other researchers (as is indicated by the authors in the submission form?

Specific comments

1. Line 349: The authors should consider adding Bennett et al Molecular and Cellular Neuroscience 41: 337-47 2009 to the list of references supporting adult neurogenesis in "non-neurogenic" regions. This paper provides good evidence for neurogenic niches in the circumventricular organs. They might also add Migaud et al European Journal of Neuroscience 32:2042-52 2010, which identifies the third ventricular region of the hypothalamus as a neurogenic niche (as does the already cited Kokoeva paper).

2. Figure 1A. I am not seeing the arrowhead that points to the TK+ stellate astrocytes. It may be missing or too small or obscured.

3. Figure 2B. Please specify which time point the images are from. I could not find a specific reference to 2B in the text, so it's not clear whether this information is given in the results section.

4. Figure 4 caption, line 572. Do the authors mean to say that TK rats consumed less (not more) sucrose? I'm looking at the middle graph in Figure 4C. And does this sentence indeed refer to the middle graph? If so, then that should be specifically indicated, as it has been for the left and right graphs in the figure.

5. Statistical table: should the header for the right-most column be p value, not power? Maybe eNeuro instructs authors to call p values power; if so, that doesn't sound right to me.

6. Typo line 66: Herpes Simplex, not Simples

7. Line 119: delete "of" after "after"

8. Line 195: delete "and" after environment (or insert "are" are mice in line 194)
